# Accuracy of atrial fibrillation detection by an insertable cardiac monitor in patients undergoing catheter ablation: Results of the BioVAD study

**DOI:** 10.1111/anec.12960

**Published:** 2022-04-28

**Authors:** Amira Assaf, Dominic A. M. J. Theuns, Rafi Sakhi, Rohit E. Bhagwandien, Tamas Szili‐Torok, Sing‐Chien Yap

**Affiliations:** ^1^ Department of Cardiology, Erasmus MC University Medical Center Rotterdam Rotterdam The Netherlands

**Keywords:** atrial fibrillation, BioMonitor III, catheter ablation, implantable loop recorder, insertable cardiac monitor, pulmonary vein isolation

## Abstract

**Background:**

Insertable cardiac monitors (ICMs) are increasingly used to evaluate the atrial fibrillation (AF) burden after catheter ablation of AF. BioMonitor III (BM3) is an ICM with a long sensing vector, which enhances sensing capabilities. The AF detection algorithm of the BM3 is based on R–R interval variability.

**Objective:**

To evaluate the performance of the AF detection algorithm of BM3 in patients before and after catheter ablation of AF using simultaneous Holter recordings.

**Methods:**

In this prospective study, we enrolled patients scheduled for catheter ablation of paroxysmal or persistent AF. After BM3 implantation, patients had a 4 days Holter registration before and 3 months after ablation. All true AF episodes ≥2 min on the Holter were annotated and matched with BM3 detected AF detections.

**Results:**

Thirty‐one patients were enrolled (mean age 60 ± 8, 74% male, 68% paroxysmal AF). Fifty‐six Holter registrations were performed in 30 patients. Twelve patients demonstrated at least one true AF episode with a total AF duration of 570 h. The AF burden accuracy of BM3 before catheter ablation was 99.6%, with a duration sensitivity of 98.6% and a duration specificity of 99.9%. The AF burden accuracy of BM3 after catheter ablation was 99.8%, with a duration sensitivity of 90.2% and a duration specificity of 99.9%. Overall, the AF burden detected on the Holter and BM3 demonstrated a high Pearson correlation coefficient of 0.996.

**Conclusion:**

BM3 accurately detects AF burden in patients before and after catheter ablation of AF.

## 1 INTRODUCTION

Insertable cardiac monitors (ICMs) are increasingly used in clinical trials to determine the efficacy of catheter ablation of atrial fibrillation (AF).(Andrade et al., 2019; Andrade et al., 2021; Perez‐Castellano et al., 2014) Continuous monitoring provides the opportunity to establish the AF burden, which may have a better correlation with functional outcome than mere documentation of AF recurrence by intermittent rhythm monitoring strategies.(Andrade et al., 2020; Sakhi et al., 2019; Sanchez‐Somonte et al., 2021) Furthermore, intermittent Holter monitoring not only has a lower sensitivity but paradoxically may also overestimate the AF burden in those with AF recurrence.(Aguilar et al., 2022) As AF burden may play a more important role in the evaluation of novel ablation techniques, it is important that the AF burden provided by ICMs is accurate. In contrast to AF episodes, the AF burden provided by the ICMs cannot be validated by adjudication of the underlying ECG. Knowledge on the performance for AF detection of ICMs from different manufacturers is, therefore, crucial. Studies have provided AF detection performance data for several ICM models, but most studies are limited to the adjudication of AF episodes detected by the ICM.(Mittal et al., 2016; Afzal et al., 2020) To estimate the sensitivity, it is also important to identify episodes of AF missed by the ICM, requiring simultaneous Holter recordings. While a few studies have done this,(Sanders et al., 2016; Piorkowski et al., 2019; Purerfellner et al., 2018; Nolker et al., 2016) we are not aware of any study that has also evaluated the diagnostic performance after catheter ablation. Following Bayes theorem, the diagnostic performance is influenced by the a priori AF risk, which is different after catheter ablation.

The aim of this study was to validate the performance of the AF detection of BioMonitor III (Biotronik, Berlin, Germany), before and after catheter ablation, in patients undergoing catheter ablation for AF. Emphasis was placed on the accuracy of duration metrics for the calculation of AF burden.

## 2 METHODS

### 2.1 Study design and population

The *BioMonitor III: Validation of the Atrial fibrillation Detecting algorithm in patients undergoing pulmonary vein isolation* (BioVAD) study was an investigator‐initiated, prospective, non‐randomized, single‐center study, conducted in the Erasmus Medical Center (Rotterdam, the Netherlands). Patients above the age of 18, who were scheduled to undergo catheter ablation of paroxysmal or persistent AF were eligible for enrolment. Patients with long‐standing persistent AF and permanent AF were excluded.

### 2.2 Study objectives

The primary objective was to determine the sensitivity, specificity, positive predictive value (PPV), and negative predictive value (NPV) of the ICM in detecting AF compared to simultaneous Holter monitoring before and after catheter ablation. The secondary objective was to describe ICM‐ or insertion‐related complications. At study entry, a BioMonitor III was implanted. After 1 week, patients received a Holter for up to 4 consecutive days. Catheter ablation was done within 3 months, and 3–5 months later, patients received a second 4 days Holter.

### 2.3 Device characteristics

The BioMonitor III is made of hermetically sealed biocompatible titanium coated in silicone.(Deneke et al., 2022) It consists of a solid housing and a flexible lead body and has a volume of 1.9 cc. Up to 56 arrhythmia episodes (60 seconds per episode) can be stored in the ICM memory. Remote monitoring is possible with the Biotronik Home Monitoring system. Every night, the ICM sends up to 6 ECG strips to a patient device (CardioMessenger, Biotronik) via radiofrequency, which relays it to the Home Monitoring Service Center via a mobile connection. The Home Monitoring website provides a secure interface to review arrhythmia episodes and further data, such as the AF burden (defined as the percentage of time in AF of the last 24 h) and the sensing performance.

### 2.4 Atrial fibrillation detection algorithm

The AF detection algorithm is based on continuous checks of the R–R interval variability according to programmable parameters. In our study, an R–R interval was defined unstable if it deviated by more than 12% from the previous. If in two consecutive windows of each 8 R–R intervals at least 5 intervals are unstable, the algorithm suspects AF and starts a confirmation period of 2 min. If within this confirmation period, in two consecutive windows of each 16 R–R intervals only 3 or less R–R intervals are unstable, the confirmation phase is terminated without AF being detected. However, if the confirmation period expires without this termination criterion fulfilled, AF is detected, and an ECG is stored. The AF episode lasts until the same termination criterion is fulfilled. All these mentioned parameters are programmable. In our study, we programmed bigeminy rejection “standard.” The detection of other arrhythmias such as high ventricular rate, bradycardia, and asystole was turned off.

### 2.5 Simultaneous Holter recordings

The AF algorithm was validated with Holter recordings of up to 96 h. LifeCard CF Holter recorders (Spacelabs Healthcare, Snoqualmie, WA, USA) were used to record two leads (lead I and II). The internal clocks of the ICM and the Holter device were synchronized at the start. Holter data were analyzed using the Pathfinder SL analysis software (version 1.7.1.5201, Spacelabs Healthcare). AF in the ECG was annotated by an experienced Holter technician blinded to ICM data and reviewed by a physician.

### 2.6 Statistical analysis

**FIGURE 1:**
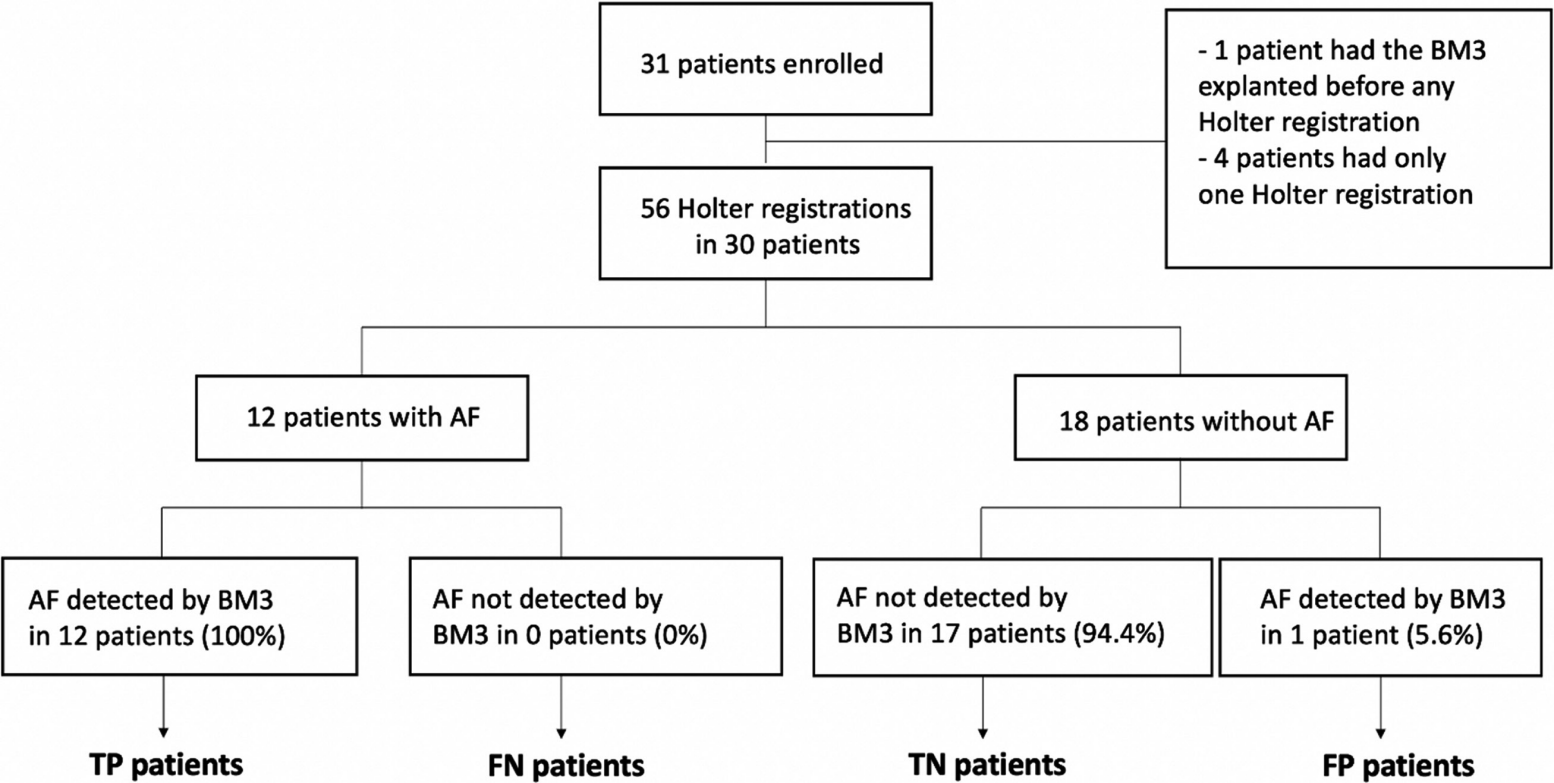
Study flow chart and diagnosis. Abbreviations: AF, atrial fibrillation; BM3, BioMonitor III; FN, false negative; FP, false positive; TN, true negative; TP, true positive

**FIGURE 2:**
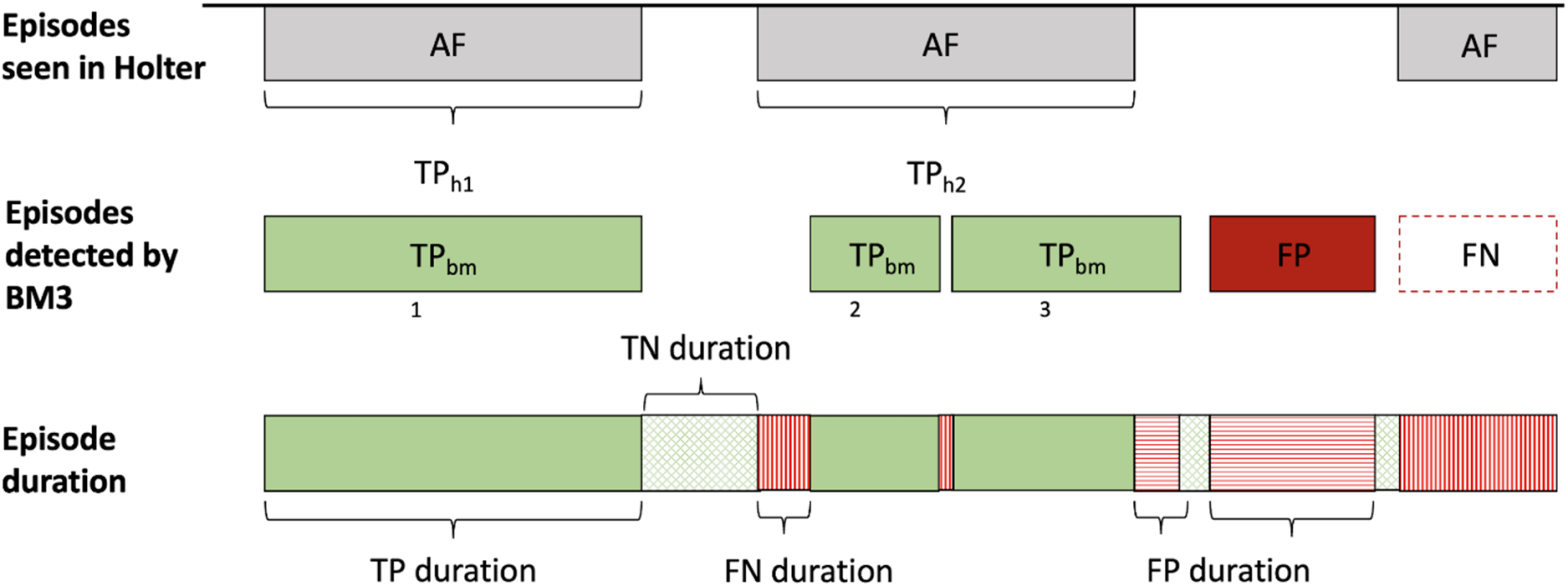
Definitions of episode and duration metrics. Abbreviations: AF, atrial fibrillation; BM3, BioMonitor 3; FN, false negative; FP, false positive; TN, true negative; TP_hX_, true positive Holter‐episode; TP_bmX_, true positive BioMonitor‐episode. TP_hx_ are AF episodes detections in the Holter that are also detected by the BM3. TP_bmX_ are AF episode detections on the BioMonitor 3 during a true AF episode according to the Holter. There might be ≥2 AF episode detections by the BM3 during a single AF episode on the Holter

AF detections by the Holter were considered the gold standard. The Holter annotations for true AF episodes of ≥2 min were compared with AF detected by the ICM. Holter segments with atrial flutter, noise, or motion artifacts were excluded from the analysis. Episode‐based metrics and duration‐based metrics were calculated for the entire duration of all Holters (Figure S1).(Sanders et al., 2016) Subject‐based metrics evaluated both Holter recordings per patient if two were recorded (Figure 1). Episode‐based metrics give the sensitivity and PPV (Figure 2), while specificity and NPV cannot be computed because a true‐negative episode is not defined. Duration‐based metrics are based on the temporal overlap of identified episodes of AF between the Holter recording and the ICM (Figure 2). The AF burden reported by the ICM was compared with the Holter‐derived true AF burden by the Pearson correlation coefficient.

## 3 RESULTS

### 3.1 Study population

We enrolled 31 patients who were scheduled to undergo catheter ablation of AF. Baseline characteristics of the study population are presented in Table 1. Twenty‐nine patients completed a Holter before catheter ablation. Two patients had no Holter before catheter ablation because of an early explantation of the device and because of technical issues with the Holter, respectively. Furthermore, three patients did not complete a Holter after catheter ablation because of patient refusal in two and logistical issues in the third. A total of 56 Holter recordings from 30patients were thus completed, with 4614 h of analyzable recordings (mean recording length per patient 82 ± 19 h). Fourteen Holter recordings (25%) in 12 patients (40%) demonstrated at least one true AF episode of ≥2 min, which yielded a total of 570 h of true AF.

**TABLE 1:** Baseline characteristics

	Enrolled subjects (*n* = 31)
Age at implantation, years	60 ± 8
Male sex	23 (74)
BMI (kg/m^2^)	28 ± 4
AF classification	
Paroxysmal AF	21 (68)
Persistent AF	10 (32)
ECG characteristics	
Normal QRS‐axis	20 (65)
Left QRS‐axis	5 (16)
PR interval (msec)	157 ± 74
QRS duration (msec)	103 ± 15
QTc interval (msec)	415 ± 30
P‐wave amplitude lead II (mV)	0.12 ± 0.07
P‐wave amplitude lead aVF (mV)	0.10 ± 0.06
R‐wave amplitude lead II (mV)	1.05 ± 0.59
R‐wave amplitude lead aVF (mV)	0.80 ± 0.58
R‐wave amplitude ICM at implantation (mV)	0.76 ± 0.41
Medication	
Class I AAD	11 (36)
Class II AAD	15 (48)
Sotalol	10 (32)
Amiodaron	3 (10)
Class IV AAD	4 (13)
Digoxin	1 (3)

Data are presented as *n* (%) or mean ± standard deviation.

Abbreviations: AAD, antiarrhythmic drugs; AF, atrial fibrillation; BMI, body mass index; ICM, insertable cardiac monitor.

### 3.2 Primary objective

#### 3.2.1 Subject‐based performance metrics

All 12 patients with AF episodes on their Holter had also detection of AF by their ICM resulting in a subject‐based sensitivity of 100% (Figure 1, Table 2). In one patient, the ICM detected AF while no AF was seen on the Holter (subject‐based specificity 94.4%). Overall, the ICM had a diagnostic accuracy of 96.7%.

**TABLE 2:** Performance metrics of BioMonitor III

	Pooled metrics 2 min	Before CA 2 min	After CA 2 min
Duration‐based results (%)			
Sensitivity	98.7	98.6	90.2
Specificity	99.9	99.9	99.9
PPV	99.3	99.6	85.7
NPV	99.8	99.6	99.9
Accuracy	99.7	99.6	99.8
Episode‐based results (%)			
Sensitivity	100	100	100
PPV	76.3	84.0	71.1
Subject‐based results (%)			
Sensitivity	100	100	100
Specificity	94.4	94.4	89.5
PPV	92.3	91.7	84.6
NPV	100	100	100
Accuracy	96.7	96.6	93.3

Abbreviations: CA = catheter ablation; PPV, positive predictive value; NPV, negative predictive value.

#### 3.2.2 Episode‐based performance metrics

All true AF episodes were all detected by the ICM (episode‐based sensitivity 100%) (Table 2). Of the 97 AF detections made by ICM, 74 were true positive (episode‐based PPV 76.3%).

#### 3.2.3 Duration‐based performance metrics

**FIGURE 3:**
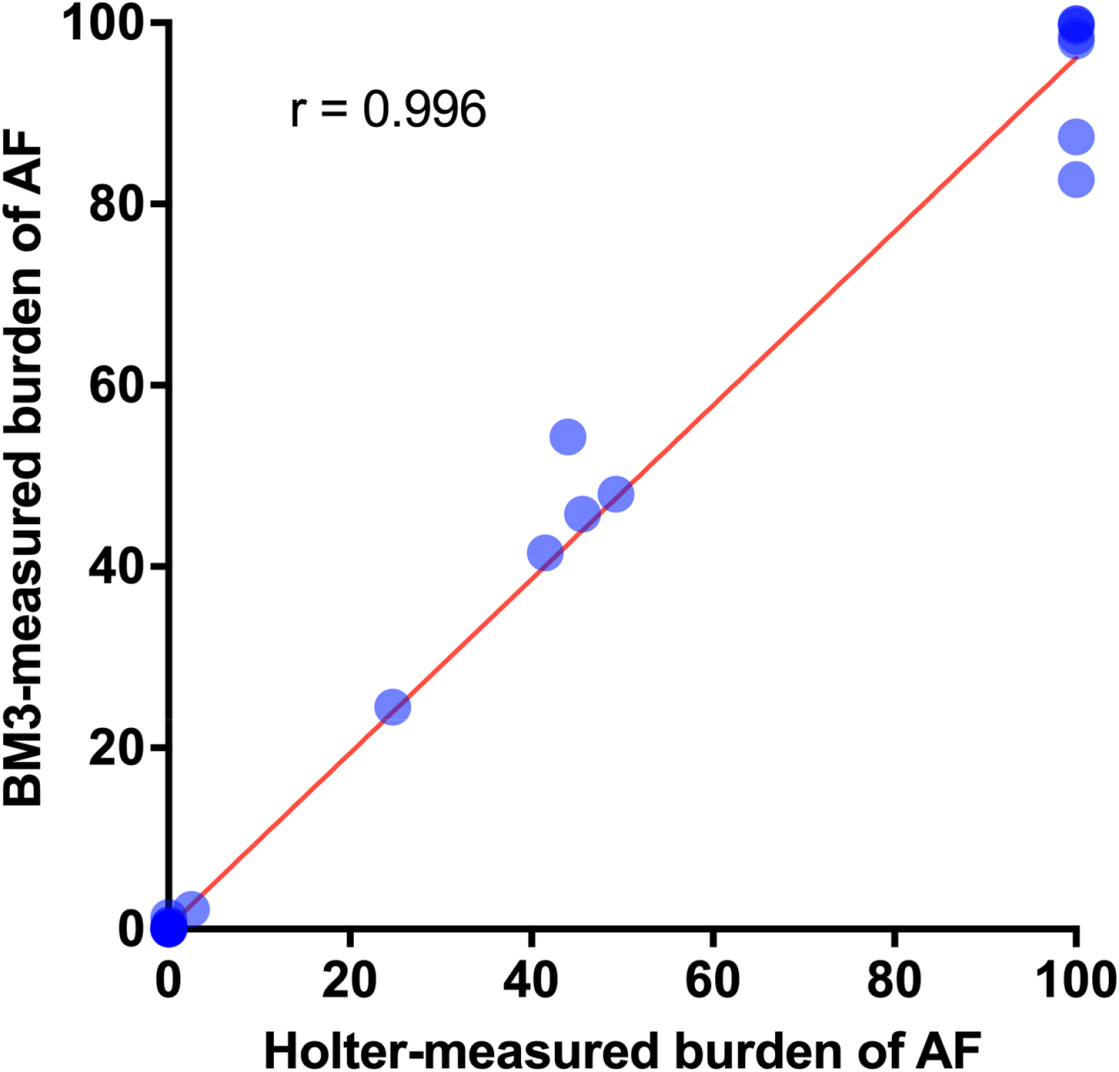
Scatterplot of AF burden detected by Holter and BioMonitor III. There was a high Pearson correlation coefficient between AF burden detected by the Holter and BioMonitor III. Abbreviations: AF, atrial fibrillation; BM3, BioMonitor III

The proportion of correctly identified episode duration was 99.7% (duration‐based accuracy), with a sensitivity of 98.7% and specificity of 99.9% (Table 2). The duration‐based PPV was 99.3%. AF burden detected on the Holter and ICM demonstrated a Pearson correlation coefficient of 0.996 (Figure 3). Although all AF episodes were correctly identified by the ICM, segments of AF episodes were rejected by the ICM algorithm due to a reduction in R–R interval variability, resulting in a duration‐based sensitivity of 98.7%. This value was especially lower after catheter ablation (Table 2).

### 3.3 Before and after catheter ablation

**FIGURE 4:**
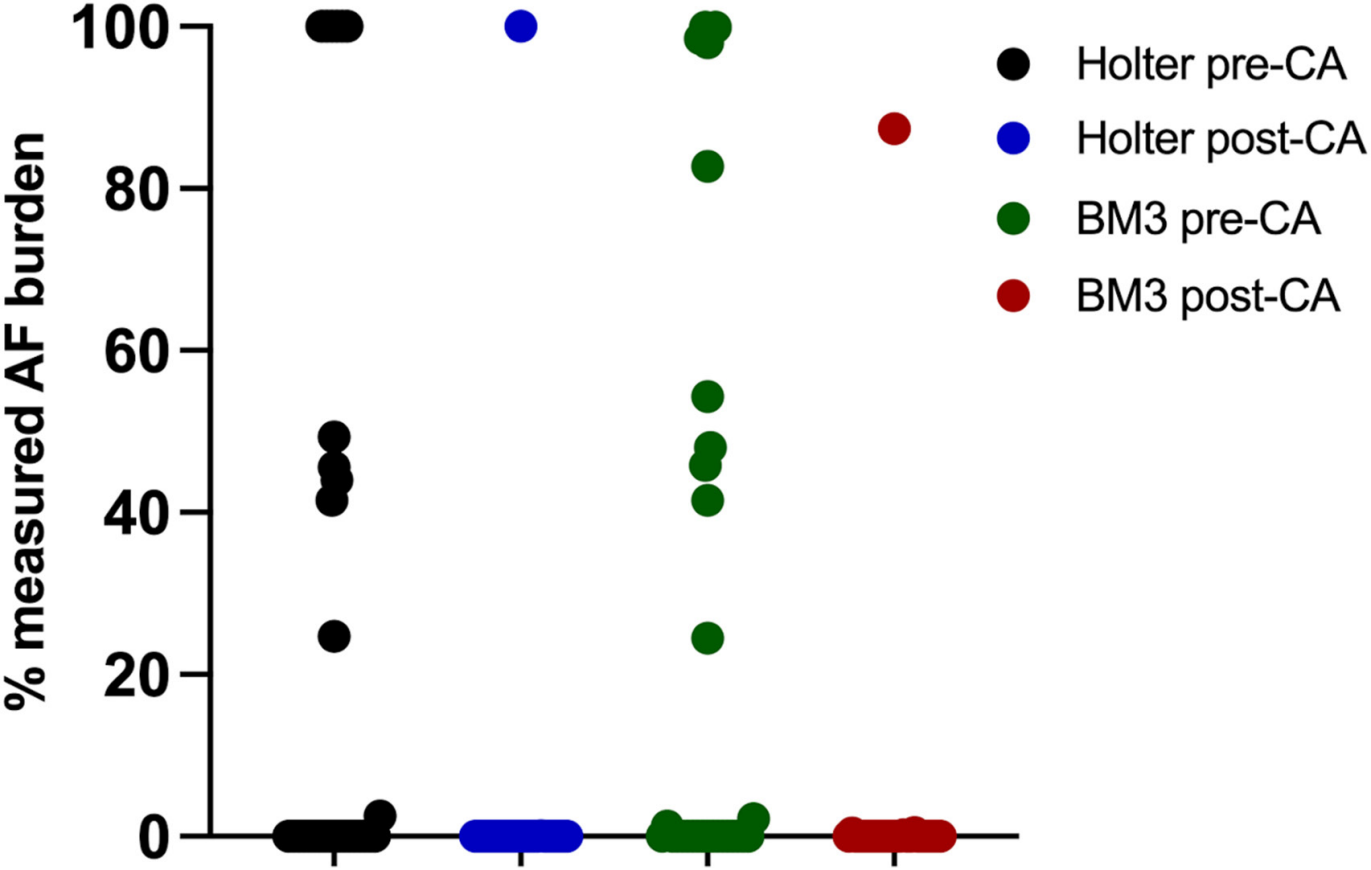
Measured AF burden before and after catheter ablation. Abbreviations: AF, atrial fibrillation; BM3, BioMonitor III; CA, catheter ablation

After catheter ablation, there was a lower AF burden in comparison to baseline (Figure 4). The total duration of true AF before catheter ablation was 522 h (21.7% of Holter recording duration) versus 12 h (0.5% of Holter recording duration) after catheter ablation (*p* < .001). When comparing the performance metrics before and after catheter ablation, most metrics remained similar (Table 2). However, there was a numerical reduction in PPV on subject, episode, and duration level. The reduction in PPV was related to a reduction in the number of true positive AF episodes detected by the ICM with a similar number of false‐positive AF episodes, supporting the assumption we made in the introduction that the incidence of AF influences the detection performance.

### 3.4 False‐positive AF detections

**FIGURE 5:**
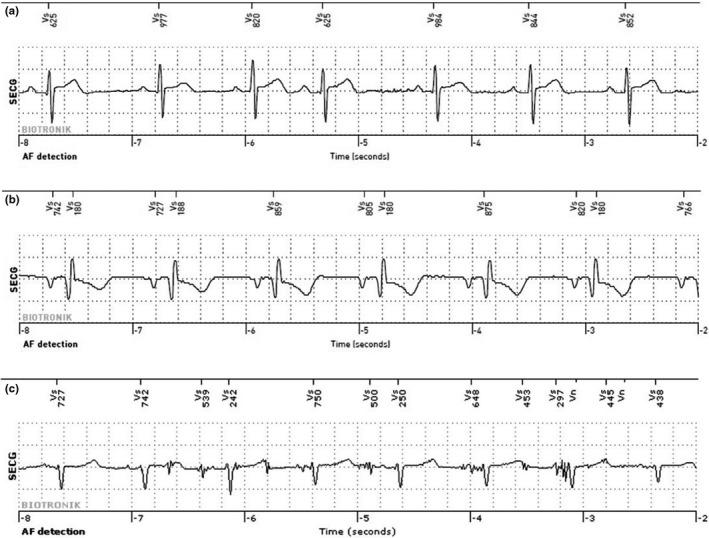
Examples of false‐positive AF detections by BioMonitor III. (a) False‐positive AF detection due to atrial ectopy. (b) False‐positive AF detection due to P‐wave oversensing (c) false‐positive AF detection due to noise

In total, there were 23 false‐positive episodes by BioMonitor III. Reasons for false‐positive episodes were atrial ectopy (*n* = 11, 48%), P‐wave oversensing (*n* = 11, 48%), and noise (*n* = 1, 4%). Examples of false‐positive detections are shown in Figure 5.

Benchmark testing of several false‐positive episodes was performed to evaluate the effect of a novel enhanced AF detection algorithm (Rhythm Check) and individualized settings. Rhythm Check is available in BioMonitor IIIm (Biotronik). It recognizes specific patterns associated with ectopy (short and long interval equal in sum to two intervals of the current rhythm) and considers them stable for the purpose of AF detection. We performed a post hoc bench test with Rhythm Check on the false detections caused by atrial ectopy. This resulted in a 78% rejection of false detections due to atrial ectopy (Table S1). Furthermore, we tested whether changing the sensing setting to “T‐wave suppression” could reduce false AF detections due to P‐wave oversensing. In fact, this reduced false AF detections by 90% (Table S1). Thus, both bench tests indicate that a good proportion of the false detections could have been avoided with the tested settings, but a proof for this assumption is impossible without replaying of a longer ECG segment than the one minute we had available.

### 3.5 Secondary endpoint

Four patients (13%) experienced any ICM‐ and or insertion‐related complication in our study. The first patient was explanted in the first week post‐implantation due to extrusion of the device and was excluded from further participation in the study. The potential cause of the extrusion was most likely a too‐small pocket size. The second patient was explanted after finishing the second Holter due to an uncomfortable feeling with the device, most probably due to the proximity of the flexible part of the device and abdominal adipose tissue. The third patient had a superficial wound infection of the pocket and was treated conservatively with antibiotics. The fourth patient had loss of signal after an external electrical cardioversion where the patches were placed directly over the device.

## 4 DISCUSSION

The main finding of the BioVAD study is that BioMonitor III provides an accurate estimate of AF burden in patients before and after catheter ablation for AF. The ICM correctly detected 98.7% of the total AF duration and 99.9% of the total normal sinus rhythm. The duration‐based accuracy was similar before and after catheter ablation (99.6% and 99.8%, respectively). This implies that the AF burden as provided by BioMonitor III can be reliably used for the follow‐up of patients after catheter ablation of AF. To the best of our knowledge, this is the first study that specifically evaluated the performance of an ICM after catheter ablation of AF.

Although the optimal outcome measure (e.g., 30 sec AF, AF burden, etc.) after catheter ablation remains to be determined,(Hindricks et al., 2021; Calkins et al., 2017) reduction in AF burden seems to be a more accurate reflection of procedural success than a single AF recurrence.(Sanchez‐Somonte et al., 2021; Verma et al., 2013) Improvement in quality of life (QOL) post‐ablation is better correlated with reduction in AF burden than AF recurrence.(Mantovan et al., 2013; Blomstrom‐Lundqvist et al., 2019; Terricabras et al., 2020) Many patients experience an improvement in QOL post‐ablation without total elimination of AF. Furthermore, baseline AF burden predicts post‐ablation outcomes better than the subjective variable of paroxysmal versus persistent AF.(Andrade et al., 2020) Therefore, continuous monitoring with ICMs will likely play an important role in the future, if an objective quantification of ablation success is required, for example, in clinical trials evaluating the ablation of AF.

BioMonitor III has a similar AF algorithm as its predecessor, BioMonitor 2, but it is smaller.(Deneke et al., 2022) Piorkowski et al. evaluated the AF detection algorithm of BioMonitor 2 in a multicenter study.(Piorkowski et al., 2019) A total of 84 patients had a Holter and corresponding ICM data. There were 15 patients (18%) with Holter‐detected AF ≥6 min with a total duration of Holter‐detected AF of 401 h. The AF duration sensitivity, duration specificity, and duration accuracy of BioMonitor 2 were 93.6%, 99.2%, and 98.7%, respectively. Our AF detection performance metrics (98.7%, 99.9%, 99.7%) appear better but there are differences between both studies regarding patient population and AF detection settings (e.g., 6 min versus 2 min AF confirmation time, standard versus individualized AF detection programming). The smaller size of BioMonitor III in comparison to its predecessor (1.9 cc versus 5 cc) does not seem to have a negative impact on the AF detection performance metrics.

The accuracy of BioMonitor III for the detection of AF is comparable to the ICMs of other manufacturers. Sanders et al. reported the AF detection performance metrics of the Reveal LINQ (Medtronic, Minneapolis, MN, USA) in 138 patients with a documented history of AF and ablation candidates.(Sanders et al., 2016) In this study, there were 38 patients (28%) with Holter‐detected AF of ≥2 min with a total duration of Holter‐detected AF of 450 h. Using R–R interval variability and a proprietary P‐wave recognition algorithm, the AF duration sensitivity, duration specificity, and duration accuracy of Reveal LINQ were 98.4%, 99.5%, and 99.4%, respectively. These data are comparable to our results (Table S1). Nölker et al. reported the AF detection performance metrics of the Confirm DM2102 (St. Jude Medical, St. Paul, MN, USA) in 79 patients with suspected or known paroxysmal AF.(Nolker et al., 2016) In this study, there were 16 patients (20%) with Holter‐detected AF of ≥2 min with a total duration of Holter‐detected AF of 636 h. AF duration sensitivity and duration specificity of Confirm DM2102 was 83.8% and 99.4%, respectively. To the best of our knowledge, there are no published AF performance data of Confirm Rx.

An important cause of false‐positive AF episodes in our study was the presence of atrial ectopy. Its successor to the device, BioMonitor IIIm (Biotronik) has a novel ectopy rejection algorithm (Rhythm Check) which we were able to use on false detections from our study. The bench testing showed a marked reduction in false‐positive AF episodes due to ectopy when Rhythm Check was applied (Table S1). This seems to be a promising feature, but bench tests must be taken with some skepticism. Future studies will have to be conducted to test this novel algorithm in clinical practice and evaluate whether this does not negatively impact the sensitivity for detecting AF.

In our study, we used fixed settings for all our study patients. Individualized AF detection settings could improve AF detection metrics. Adjustment of the sensing high pass filter, target sensing threshold, or SensingConsult are some of the possibilities. The long sensing vector of BioMonitor III improves the visibility of P‐waves; however, this also resulted in false‐positive AF episodes due to P‐wave oversensing in some patients. Changing the SensingConsult to T‐wave suppression can reduce oversensing of P‐waves (Table S1). Furthermore, in some patients it may be necessary to reduce the R‐R variability limit to a lower number to detect more regular AF.

In future devices, incorporation of an artificial intelligence (AI) filter might be promising. Mittal et al. studied if an AI filter improves the AF detection performance of Reveal LINQ.(Mittal et al., 2021) The AI filter was able to remove 66% of all false‐positive AF episodes. The improvement was the greatest for shorter episodes (<30 min). However, currently, an AI filter seems to be computationally too demanding to be embedded in existing ICMs.

### 4.1 Study limitations

Several limitations should be pointed out. First, our study population was relatively small in comparison to other validation studies.(Sanders et al., 2016; Piorkowski et al., 2019; Purerfellner et al., 2018; Nolker et al., 2016) However, the total duration of Holter recording (>4500 h including 570 h of true AF) was relatively long, which renders the duration metrics relatively reliable. Second, the study protocol required that all patients used similar AF detection settings. In clinical practice, individualized settings are more common. We speculate that the performance metrics of BioMonitor III would improve if individualized settings were used. However, to provide robust and reproducible metrics all patients in our study had to use identical settings.

## 5 CONCLUSIONS

The AF detection performance of BioMonitor III is good, with a very high duration sensitivity and specificity, thereby providing reliable estimates of the AF burden before and after catheter ablation. Performance metrics could potentially be further improved by enhanced AF detection algorithms, which reject ectopy, and by applying individualized AF detection settings.

## CONFLICTS OF INTEREST

None.

## ETHICAL APPROVAL

This study was conducted in accordance with the Declaration of Helsinki and the international standard for clinical investigation of medical devices in human subjects, ISO 14155. The local ethics committee approved the study (Netherlands Trial Register, NL7777), and all patients gave written informed consent.

## AUTHOR CONTRIBUTIONS

AA, RS, and SCY designed the study. AA performed device implantations, data collection, and statistics. AA and SCY drafted the article. All the authors performed data analysis and critically revised the article, have read and accepted the final version of the article and take responsibility for the work, and meet ICMEJ criteria for authorship.

## Supporting information


Appendix S1

## Data Availability

The data that support the findings of this study are available from the corresponding author upon reasonable request.
