# Supplementary material for: Accuracy of atrial fibrillation detection by an insertable cardiac monitor in patients undergoing catheter ablation: Results of the BioVAD study
Source: Ann Noninvasive Electrocardiol. 2022 Apr 28;27(3):e12960. doi: 10.1111/anec.12960 (PMC9107077; doi:10.1111/anec.12960)
Supplement: Supplementary file 1 — Appendix S1 [file ANEC-27-e12960-s001.docx]

**Appendix**

**Supplemental figure S1.** Calculation of subject-, episode- and duration-based performance metrics.


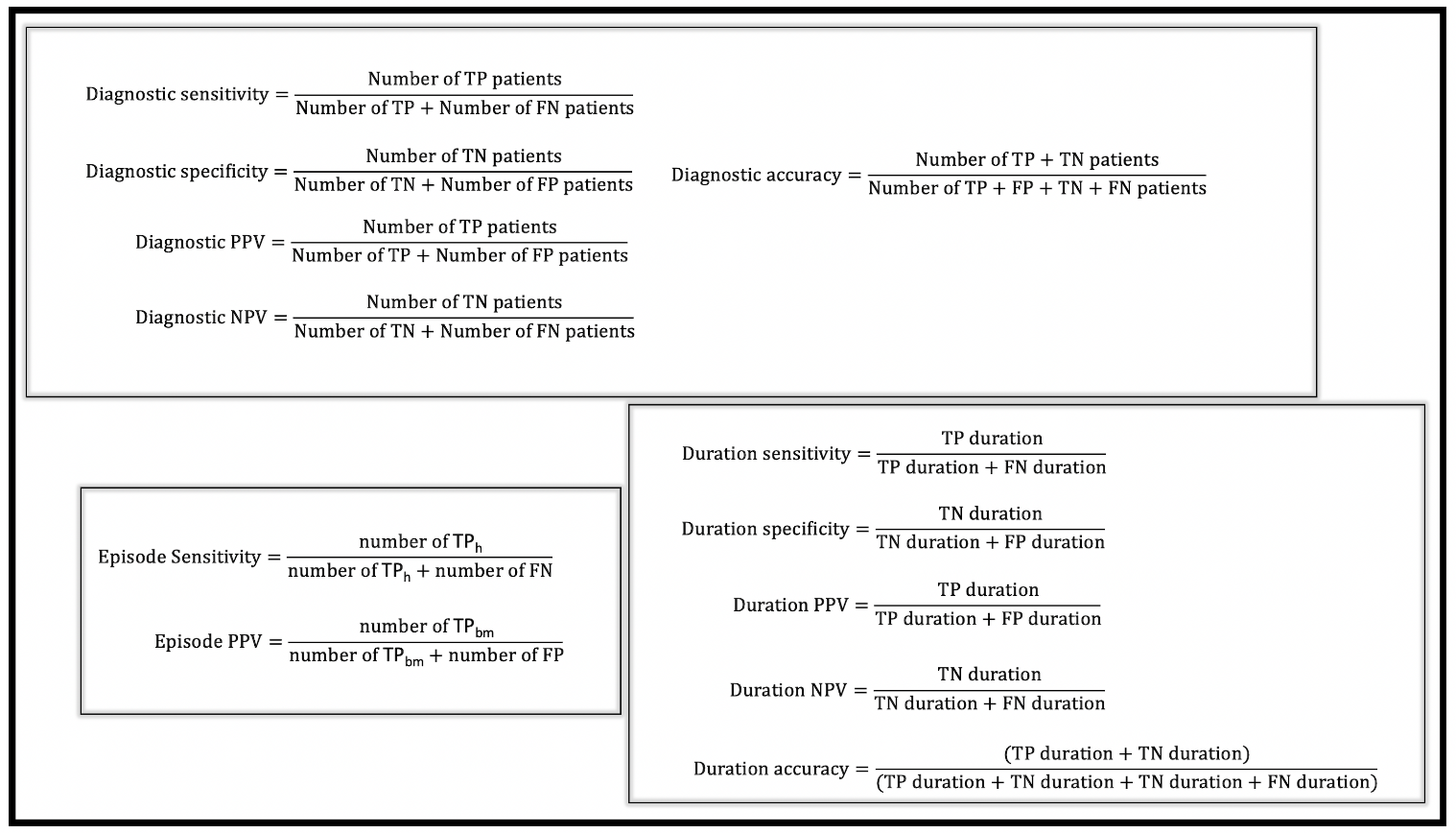


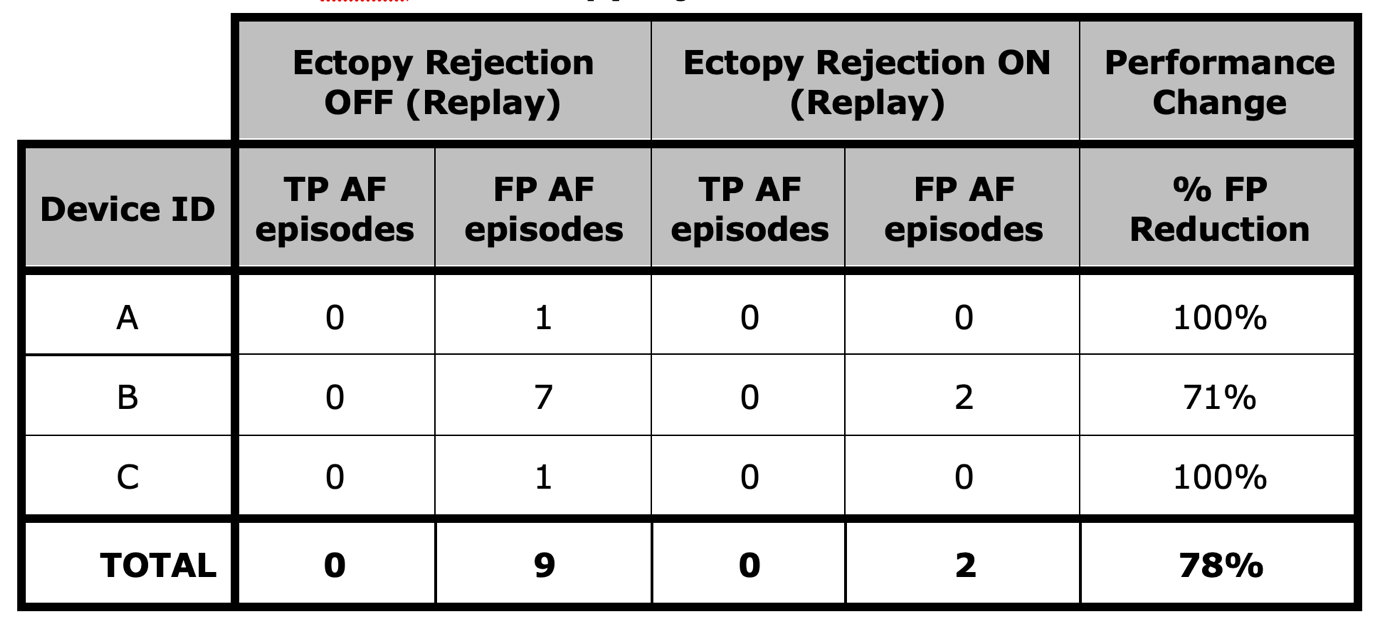
**Supplemental table S1.** Evaluation of the effect of the ectopy rejection feature in BioMonitor IIIm in patients with false AF detections due to ectopy. The ectopy rejection feature reduced the number of false AF detections due to ectopy by 78%.

**Supplemental table S2.** Evaluation of the effect of changing the SensingConsult settings in patients with false AF detections due to P-wave oversensing by BioMonitor III. Changing the SensingConsult from “standard” to “T-wave suppression” reduced false AF detections due to P-wave oversensing by 90%.

**
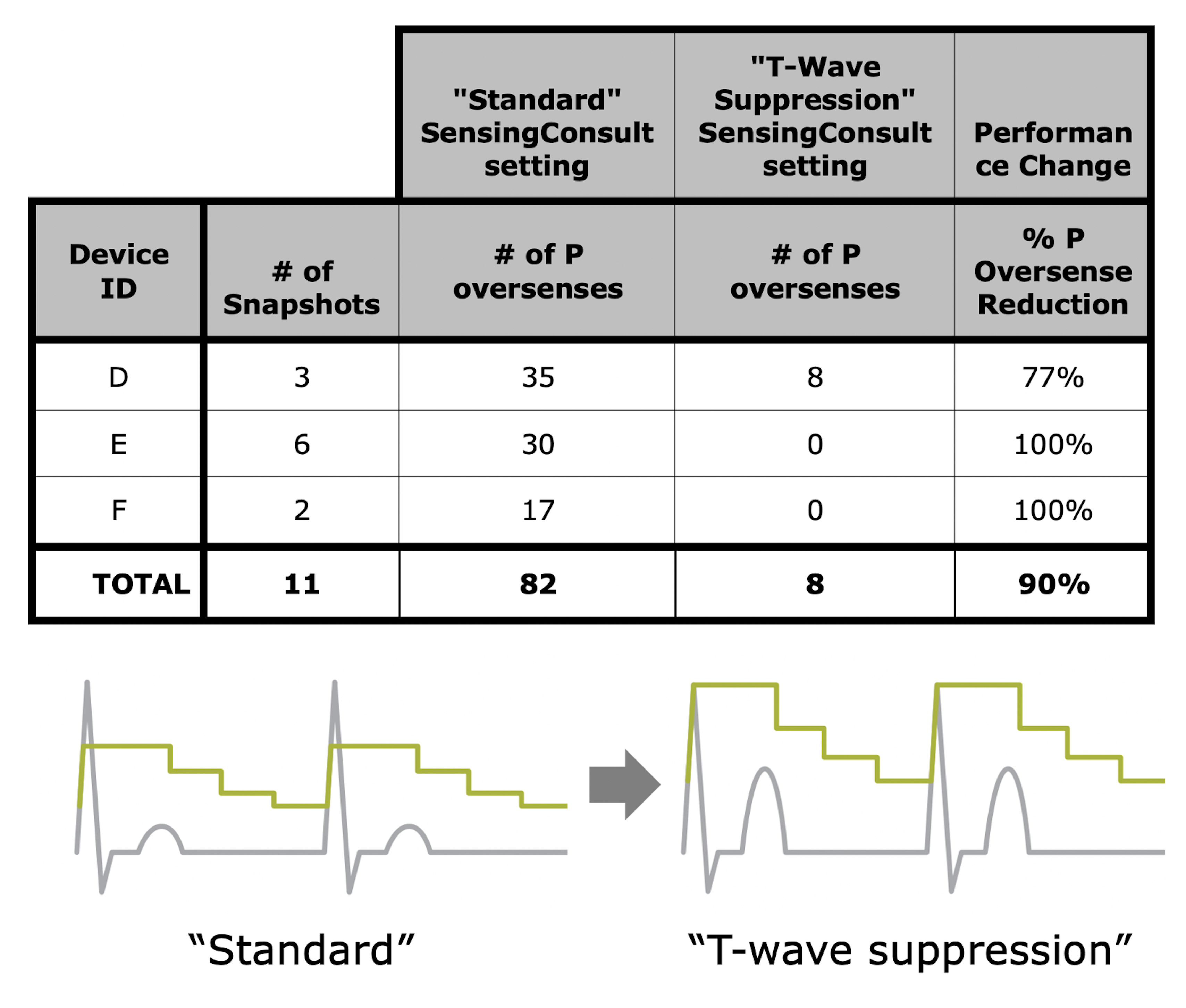
**

**Supplemental table S3.** Comparison of the performance metrics of BioMonitor III versus Reveal LINQ for the detection of AF.

| **Device** | **BioMonitor III**  (BioVAD study)^†^ | **Reveal LINQ**  (Sanders et al. 2016)^‡^ |
| --- | --- | --- |
| **Duration-based results (%)** |  |  |
| Sensitivity | 98.6 | 98.4 |
| Specificity | 99.9 | 99.5 |
| PPV | 99.6 | 97.2 |
| NPV | 99.6 | 99.7 |
| Accuracy | 99.6 | 99.4 |
| **Episode-based results (%)** |  |  |
| Sensitivity | 100 | 97.3 |
| PPV | 84.0 | 74.8 |
| **Subject-based results (%)** |  |  |
| Sensitivity | 100 | 97.4 |
| Specificity | 94.4 | 97.0 |
| PPV | 92.4 | 92.5 |
| NPV | 100 | 99.0 |
| Accuracy | 96.7 | 97.1 |

Abbreviations: NPV, negative predictive value; PPV, positive predictive value. ^†^ Only the metrics before catheter ablation were used as the study of Sanders et al. included patients with a documented history of AF and ablation candidates.^10^ ^‡^ The AF detection algorithm in the study of Sanders et al. was based on R-R interval variability and P-wave evidence score.^10^
